# A Dangerous Diet

**Published:** 1886-02

**Authors:** 


**A Dangerous Diet.**—The Los Angeles *Herald*, of November 20th, says, At nearly midnight last night word was brought to the Sheriff's office in this city that a man had been found in the lower part of San Fernando valley in an almost dying condition from having eaten the fruit of the cactus, or tunas. The mouth, tongue and throat were terribly swollen from the poison from the spines, which are as fine as hair and sharp as needles. They are barbed, too, and when once they have penetrated the flesh it is all but impossible to extract them. The Indians eat them, but they are skilled in removing the spines in the most careful manner.

**Dr. Wood** says that Bright's disease of the kidneys is very largely attributed to the use of alcohol, an undoubted truth.

